# Update on antibiotic resistance in foodborne *Lactobacillus* and *Lactococcus* species

**DOI:** 10.3389/fmicb.2013.00301

**Published:** 2013-10-08

**Authors:** Chiara Devirgiliis, Paola Zinno, Giuditta Perozzi

**Affiliations:** CRA-NUT, Food and Nutrition Research Center, Agricultural Research CouncilRoma, Italy

**Keywords:** AR genes, growth promoters, lactic acid bacteria, fermented food, dairy fermentation, microbiota, horizontal gene transfer

## Abstract

Lactobacilli represent a major Lactic Acid Bacteria (LAB) component within the complex microbiota of fermented foods obtained from meat, dairy, and vegetable sources. Lactococci, on the other hand, are typical of milk and fermented dairy products, which in turn represent the vast majority of fermented foods. As is the case for all species originating from the environment, foodborne lactobacilli and lactococci consist of natural, uncharacterized strains, whose biodiversity depends on geographical origin, seasonality, animal feeding/plant growth conditions. Although a few species of opportunistic pathogens have been described, lactobacilli and lactococci are mostly non-pathogenic, Gram-positive bacteria displaying probiotic features. Since antibiotic resistant (AR) strains do not constitute an immediate threat to human health, scientific interest for detailed studies on AR genes in these species has been greatly hindered. However, increasing evidence points at a crucial role for foodborne LAB as reservoir of potentially transmissible AR genes, underlining the need for further, more detailed studies aimed at identifying possible strategies to avoid AR spread to pathogens through fermented food consumption. The availability of a growing number of sequenced bacterial genomes has been very helpful in identifying the presence/distribution of mobile elements associated with AR genes, but open questions and knowledge gaps still need to be filled, highlighting the need for systematic and datasharing approaches to implement both surveillance and mechanistic studies on transferability of AR genes. In the present review we report an update of the recent literature on AR in lactobacilli and lactococci following the 2006 EU-wide ban of the use of antibiotics as feed additives in animal farming, and we discuss the limits of the present knowledge in evaluating possible risks for human health.

## Lactic acid bacteria in food

Lactic Acid Bacteria (LAB) are integral components of fermented foods, where they carry out primary and secondary fermentations leading to the final, processed products (Caplice and Fitzgerald, 1999; Rattanachaikunsopon and Phumkhachorn, 2010). Their long history of safe use in food production earned most LAB species the GRAS (Generally Regarded As Safe) designation by the US Food and Drug Administration (FDA) and the Qualified Presumption of Safety (QPS) classification by the European Food Safety Authority (EFSA) (Adams and Marteau, 1995). Different genera and species of LAB characterize the complex fermented food microbiota, with distinguished profiles in each food that depend on environmental (latitude, pedoclimatic conditions, seasonality, animal feeding, etc.) and food processing features (processing temperature and pH, length of seasoning, etc.). The probiotic hype of the past decade has led to extensive characterization of the nutritional and health-associated features of LAB, especially of lactobacilli and bifidobacteria which are highly represented in the human gut, mainly to substantiate health claims of commercial probiotic products (Parvez et al., 2006; Gonzalez-Rodriguez et al., 2013). Due to the presence of a wide variety of LAB species associated with health-promoting features, fermented foods are often considered “naturally functional.” However, the role of LAB as reservoir of antibiotic resistance (AR) determinants with transmission potential to pathogenic species is now increasingly acknowledged (reviewed in: Teuber et al., 1999; Marshall et al., 2009; van Reenen and Dicks, 2011), thus representing a potential health risk which was neglected for a long time.

Taxonomic biodiversity of the environmentally derived food fermenting LAB community, unique for each product especially in raw milk artisanal cheeses, makes it extremely difficult to attribute the emergence and spread of AR through the food chain to specific genera/species. Moreover, a detailed overall picture has not yet emerged of the genetic exchanges driving this process, especially when it relates to distinguishing between intrinsic AR (typical of all strains of a given species and non horizontally transmissible) and acquired AR (present in only few strains of a typically susceptible species and acquired by mutation or lateral gene transfer) (Klare et al., 2007; Ammor et al., 2008a; van Reenen and Dicks, 2011). In the present review we will focus on horizontally transmissible AR, which attributes to LAB the role of a “silent” reservoir of resistance.

The most relevant LAB in fermented foods belong to the genera *Lactococcus, Streptococcus, Pediococcus, Leuconostoc*, and *Lactobacillus*. Several LAB species are also highly represented within the resident gut microbiota of healthy humans. *Lactobacillus* species, in particular, are abundant in both food and gut microbiota, several strains are widely employed as probiotic supplements, and this genus includes therefore important players in genetic exchanges between the transient (foodborne) and resident colonizers of human and animal gut (Devirgiliis et al., 2011). Another important genus of LAB for food technology is represented by *Lactococcus*, with some species especially abundant in milk and fermented dairy products, which in turn constitute the vast majority of fermented foods (van Hylckama Vlieg et al., 2006). Both lactobacilli and lactococci are mostly composed of non-pathogenic species, but rather display probiotic features and were never considered a threat for humans. However, AR strains have been increasingly described following the recognition of LAB as reservoir of AR genes horizontally transmissible to pathogens through the food chain (Teuber et al., 1999; Marshall et al., 2009). A growing body of literature is now available on this issue in isolates deriving from various environmental sources. In the present review we have focused on updating the available knowledge on phenotypic AR and horizontal transmission routes of AR genes specifically in foodborne lactobacilli and lactococci. We chose to consider reports published in the past 5 years, as this time span should better reflect the situation following the 2006 EU-wide ban of the use of antibiotics as feed additives in animal farming [European Parliament and Council Regulation (EC) No. 1831/2003].

## Antibiotic resistance and horizontal gene transfer in bacteria

The spread AR in bacteria is strictly linked to the mechanisms of horizontal gene transfer (HGT), which are unrelated to vertical gene exchanges from parental generation to the offspring that occur during sexual or asexual reproduction. Also termed lateral gene transfer, HGT has been shown to represent a crucial factor in evolution, and growing awareness indicates that it could act as a major mechanism for genetic transfer amongst unicellular organisms (Alekshun and Levy, 2007).

Mobile elements (plasmids, transposons and integrons) are key players in bacterial HGT. These highly organized transferable elements often include AR genes and they are mostly responsible for intra- and inter-species transfer of genetic material (Alekshun and Levy, 2007; van Reenen and Dicks, 2011; Santagati et al., 2012). Full genome sequencing projects (Liu et al., 2005; O'Sullivan et al., 2009), as well as the application of DNA-based technologies to Gram-positive bacteria, are starting to provide a general picture of the elements conferring capacity for HGT in LAB, with mechanisms that appear to be evolutionarily similar to those identified in Gram-negatives (Thomas and Nielsen, 2005; van Reenen and Dicks, 2011). Comparative genomics, on the other hand, is providing clues as to the time-scale of horizontal gene fluxes that shaped LAB genomes during adaptation to the environment (Pfeiler and Klaenhammer, 2007). In the case of AR spreading, which originates from self-protection in antibiotics-producing bacteria (D'Costa et al., 2011), conjugation appears to be the prevalent mechanism, acting through conjugative transposons located on the chromosome or on plasmids and carrying single or multiple genes encoding resistance to a diverse array of antibiotics (Wozniak and Waldor, 2010). Transposon-mediated inter-species genetic transfer was recently described as the most frequent mechanisms contributing to AR spread in bacteria (Wozniak and Waldor, 2010). A variety of AR determinants associated with the Tn*916*-*1545* family of transposons have been described in foodborne bacteria (Hummel et al., 2007; Devirgiliis et al., 2009; Rizzotti et al., 2009). The best characterized member of this family is Tn*916*, an 18 kb conjugative transposon carrying the *tet*(M) gene and displaying broad host range toward both Gram-positive and Gram-negative bacteria (Clewell et al., 1995).

The presence of insertion sequences (IS) within bacterial genomes is also an indicator of HGT. IS are simple mobile elements, capable of autonomous transposition and often identified in association with AR genes. They are constituted by small segments of DNA flanked by short repeated sequences required for transposition, and encode only few functions involved in their own mobility (Mahillon and Chandler, 1998). Like transposons, IS elements have been found on the chromosome, on plasmids or on both, but their horizontal transfer occurs only when they are associated with conjugative elements. IS-encoded transposases promote the formation of circular elements as transient replication intermediates, which can either integrate at different chromosomal locations or be horizontally transferred to other cells (Churchward, 2002).

HGT can also occur by transduction promoted by viruses and bacteriophages (Rohwer et al., 2009). Phage-mediated transfer of AR genes has been successfully tested between different *Enterococcus faecalis* strains (Yasmin et al., 2010) as well as among enterococcal species (Mazaheri Nezhad Fard et al., 2011). Increasing interest in bacteriophages specific for Gram-positive hosts has recently stimulated investigations on their possible role as transducers of genetic information also among LAB species (Ventura et al., 2011).

The main threat associated with AR in non-pathogenic, commensal bacteria is therefore the risk of horizontal transfer of resistance determinants to human/animal pathogens, thus impairing successful antibiotic treatment of common microbial infections. The intestinal microbiota of humans and animals comprises more than 1000 bacterial species (Bik, 2009), including opportunistic pathogens capable of acquiring virulence genes, such as enterococci (Ogier and Serror, 2008). In the recent past, most published reports on selection and dissemination of AR genes within the complex bacterial community of the human gut were mainly focused on clinically relevant species (Franz et al., 2003). However, the role of foodborne bacteria is now emerging as reservoir of AR genes potentially transferable to human pathogens through HGT (Mathur and Singh, 2005).

## Relevant antibiotics in foodborne AR selection

The use of antibiotics as growth promoters in livestock and their role in selecting AR bacteria have been extensively reviewed elsewhere (Butaye et al., 2003; Wegener, 2003; Kazimierczak et al., 2006; Landers et al., 2012). Antibiotics have been used for decades in animal farming at sub-therapeutic doses as growth promoters, until this practice was recognized in the early ‘80s as a major determinant contributing to selection of AR strains in the gut of treated livestock, and to their subsequent release in the environment. Food products derived from animals colonized by AR bacteria represent therefore a transmission vehicle of AR to humans (Gonzalez-Zorn and Escudero, 2012). The circumstantial cause-effect relationship between the use of antimicrobials for growth promotion in livestock and the corresponding increase of antibiotic resistance in foodborne bacteria (Wegener, 2003), progressively led to a precautionary ban of their use as feed additives in several European countries, especially for those employed in human therapy and for their veterinary analogs. However, spread of AR bacterial species in the environment had already occurred, as shown by the steadily increasing number of reports on the presence of foodborne AR bacterial strains (Landers et al., 2012). While pathogenic species, mostly Gram negative, represent an immediate threat to human/animal health due to their difficult eradication when carrying AR determinants, AR Gram positives consist of mostly non-pathogenic species or opportunistic pathogens. Among them, several LAB species are present in the raw materials employed in fermented food production (milk, meat, vegetables), and they play a crucial role in food fermentations by acting as natural starters of environmental origin.

We provide here a brief description of the main classes of antibiotics which have elicited selection of AR strains in lactobacilli and lactococci, their use as growth promoters in livestock and the corresponding AR frequency observed in foodborne isolates. These antibiotics are comprehensively listed in Table 1, with the corresponding number of articles considered in this review in which each antibiotic has been used for selection of AR *Lactobacillus* and *Lactococcus* foodborne isolates.

**Table 1:** **List of antibiotics used for the identification of foodborne ARLb and ARLc**.

**Pharmacological class**	**Antibiotic used in “n” articles discussed in the text for Lb and Lc**			**Target**	**Mechanisms of resistance**
		**Lb**	**Lc**		
**Tetracyclines**	**Chlorotetracycline**	1	–	Ribosome	Target protection
	***Tetracycline***	14	10		Efflux
					Enzymatic inactivation
**Macrolides**	*Erythromycin*	13	5	Ribosome	Target site alterations (methylases)
	Roxithromycin	1	–		Efflux
					Enzymatic inactivation
**Glycopeptides**	Linozolid	2	–	Cell wall	Target site mutations (reduction of vancomycin binding affinity by substitution of a terminal D-lactate or D-serine for D-alanine)
	*Vancomycin*	5	4		
**Polymyxins**	**Bacitracin**	2	–	Cell membrane	Target site mutations
	Colistin	1	–	(LPS)	Efflux
					Enzymatic inactivation (rare)
**Streptogramins**	Quinupristin/dalfopristin	1	–	Ribosome	Target site alterations (methylases)
					Efflux Enzymatic inactivation
Aminoglycosides	Amikacin	1	–	Ribosome	Enzymatic inactivation
	Apramycin	1	–		Modification of cell permeability
	Gentamycin	7	2		Target site mutations (alterations at the ribosomal binding sites)
	Kanamycin	4	2		
	Neomycin	2	–		
	Spectinomycin	1	–		
	*Streptomycin*	7	3		
Beta-lactams	Amoxicillin	2	1	Cell wall	Enzymatic inactivation (β -lactamase)
	*Ampicillin*	6	3		Target site mutations (altered penicillin-binding proteins)
	Imipenem	1	1		Modification of cell permeability
	Oxacillin	1	1		Efflux
	Penicillin	3	1		
	Cloxacillin	–	1		
Cephalosporins^a^	Cephalothin	2	1	Cell wall	*see beta-lactams*
	Cefpodoxime	1	–		
	Cefsulodin	1	–		
	Ceftiofur	1	–		
	Cephalexin	–	1		
Chloramphenicol	*Chloramphenicol*	8	2	Ribosome	Enzymatic inactivation (mainly acetylases, phosphotransferases)
					Efflux
					Target site mutations
					Modification of cell permeability
Fusidanes	Fusidic Acid	1	–	Ribosome	Target site mutations (alteration of elongation factor G)
					Modification of cell permeability
Lincosamides	*Clindamycin*	5	3	Ribosome	Target site alterations (methylases)
	Lincomycin	1	–		Efflux
					Enzymatic inactivation
Quinolones	Ciprofloxacin^b^	4	1	DNA gyrase,	Target site mutations
	Nalidixic Acid	2	–	DNA	Efflux
				topoisomerase	Modification of cell permeability
					Lower target expression levels
					Target protection
					Enzymatic inactivation
Rifamycins	Rifampin	2	1	RNA polymerase	Target site mutations
					Target duplication
					Target protection
					Enzymatic inactivation
					Modification of cell permeability
Sulfonamides	Sulphamethoxazole	2	–	Dihydropteroate	Target site mutations
	Trimethoprim^c^	3	1	Synthetase (DHPS)^d^	Plasmid-borne alternative drug-resistant variants of DHPS

Antibiotics and pharmacological classes used in the past as growth promoters are indicated in bold. Those most frequently employed in the cited articles are italicized.

a Often considered a sub-class of beta-lactams.

b Fluoroquinolone.

c Diaminopyrimidine, similar to Sulphonamides.

d Enzyme involved in folate synthesis.

Tetracyclines have been extensively used as growth promoters in the ‘60s and ‘70s, (Wegener, 2003) and the corresponding resistance determinants are the most frequently described AR in foodborne LAB (Roberts, 2005; Thaker et al., 2010; Devirgiliis et al., 2011). Several genes have been identified conferring tetracycline resistance through different mechanisms (see Table 2 for gene list and corresponding references). Tet resistance genes are also highly mobilizable, due to association with known transposable elements (Clewell et al., 1995; Rice, 1998). Such associations have been frequently reported also for erythromycin resistance genes, which are also among the most widespread AR determinants in foodborne LAB (Mathur and Singh, 2005; Ammor et al., 2007).

**Table 2:** **Foodborne *Lactobacillus* species reported to carry AR genes and sources of isolation**.

**Species**	**Food sources**	**Resistance gene(s)**	**Association with mobile elements**	**Horizontal transfer**	**References**
*L. acidophilus*	Dairy	*erm*(B)	nd^a^	no	Nawaz et al., 2011
*L. animalis*	Vegetable	*erm*(B)	nd	no	Nawaz et al., 2011
		*tet*(M)	nd	nd	
*L. brevis*	Dairy, Vegetable	*tet*(M), *tet*(S)	nd	yes (*tet*(M))	Nawaz et al., 2011
	Meat	*tet*(M)	nd	nd	Zonenschain et al., 2009
		*erm*(B)	nd	nd	
*L. casei*	Dairy	*aph*(3′)-III, aadA, aadE	nd	nd	Ouoba et al., 2008
*L. curvatus*	Meat	*tet*(M)	nd	nd	Zonenschain et al., 2009
		*tet*(W)	nd	nd	
		*erm*(B)	nd	nd	
*L. delbrueckii subsp. bulgaricus*	Dairy	*tet*(M)	nd	nd	Zhou et al., 2012
		*aph*(3′)-III	nd	nd	
		*ant*(6)	nd	nd	
*L. fermentum*	Dairy	*erm*(B)	nd	yes	Nawaz et al., 2011
	Dairy	*tet*(K)	nd	nd	Thumu and Halami, 2012
		*tet*(L)	nd	nd	
		*erm*(B)	nd	nd	
		*msr*C	nd	nd	
*L. kefiri*	Dairy	*tet*(S)	nd	no	Nawaz et al., 2011
*L. paracasei*	Meat	*tet*(M)	nd	nd	Zonenschain et al., 2009
		*erm*(B)	nd	nd	
	Dairy	*tet*(M), *erm*(B)	nd	nd	Huys et al., 2008
		*tet*W, *erm*(B)	nd	nd	
		*tet*(M)	nd	nd	
	Dairy	*tet*(M)	Tn*916*	yes	Devirgiliis et al., 2009
	Dairy, meat	*tet*(M)	nd	nd	Comunian et al., 2010
		*tet*(M), *erm*(B)	nd	nd	
		*tet*(W)	nd	nd	
		*tet*(W), *erm*(B)	nd	nd	
*L. plantarum*	Dairy	*erm*(B), *tet*(M)	nd	no	Nawaz et al., 2011
	Vegetable	*tet*(M)	nd	yes	
		*tet*(S)	nd	no	
	Dairy	*tet*(M)	nd	nd	Zago et al., 2011
	Meat	*tet*(M)	nd	nd	Zonenschain et al., 2009
		*tet*(W)	nd	nd	
		*tet*(S)	nd	nd	
		*erm*(B)	nd	nd	
		*erm*(C)	nd	nd	
	Dairy	*van*(X)	nd	nd	Liu et al., 2009
	Dairy	*tet*(W)	nd	nd	Thumu and Halami, 2012
		*tet*(L)	nd	nd	
		*erm*(B)	nd	nd	
	Dairy	*erm*(B)	plasmid	yes	Feld et al., 2009
*L. reuteri*	Meat	*tet*(M)	nd	nd	Zonenschain et al., 2009
		*erm*(B)	nd	nd	
	Milk	*tet*(W)	nd	nd	Egervarn et al., 2009
	Meat	*tet*(W)	nd	nd	Thumu and Halami, 2012
		*erm*(B)	nd	nd	
*L. rhamnosus*	Meat	*tet*(W)	nd	nd	Zonenschain et al., 2009
		*erm*(B)	nd	nd	
*L. sakei*	Meat	*tet*(M)	nd	nd	Zonenschain et al., 2009
		*tet*(W)	nd	nd	
		*erm*(B)	nd	nd	
	Dairy	*tet*(M)	transposon	nd	Ammor et al., 2008b
		*tet*(L)	plasmid	nd	
*L. salivarius*	Vegetable	*erm*(B), *tet*(M)	nd	yes (ermB)	Nawaz et al., 2011
	Dairy	*tet*(M)	nd		
	Meat	*tet*(M)	nd	nd	Thumu and Halami, 2012
		*tet*(W)	nd	nd	
		*tet*(O)	nd	nd	
		*tet*(L)	nd	nd	
		*erm*(B)	nd	nd	
*L. vaginalis*	Dairy, Vegetable	*erm*(B)	nd	no	Nawaz et al., 2011

a not determined.

Erythromycin belongs to a class of antibiotics known as macrolides, which have been intensively used in the past as growth promoters (tylosin and spiramycin), together with streptogramins (virginiamycin), glycopeptides (avoparcin), evernimicins (avilamycin), and bacitracin. High frequency of AR was demonstrated to arise in bacteria toward all of these pharmacological classes (Wegener, 2003). Furthermore, cross-resistance was demonstrated in some strains toward macrolides, lincosamides, and streptogramins (MLS), due to the overlapping ribosomal binding sites of these antibiotics (Leclercq, 2002). Three streptogramins have been used either as therapeutics or for growth promotion: virginiamycin (employed in human and veterinary medicine, as well as in animal growth promotion), pristinamycin, and quinupristin/dalfopristin (derived from pristinamycin and recently introduced in human medicine). Resistant isolates have been detected mainly in *Enterococcus* species (Butaye et al., 2003), although a growing body of literature describes LAB species displaying MLS resistant phenotype [(Roberts, 2008) and references therein].

As for glycopeptides, association between the use of avoparcin in swine and poultry and increase in Glycopeptide Resistant Enterococci (GRE) is one of the best studied examples of the impact on human health of antimicrobials as growth promoters (Bager et al., 1997; Wegener, 2003). Subsequent reports of clinical isolates of vancomycin resistant *E. faecium* causing nosocomial infections represented indeed the first circumstantial evidence of a direct cause-effect relationship between antibiotics use in animal farming and the outbreak of AR in human pathogens (Bates, 1997). It should be pointed out that, in the case of vancomycin, several *Lactobacillus* species display intrinsic resistance (Nelson, 1999; Mathur and Singh, 2005) whose genetic context has not been described, other than showing that it lacks capability for horizontal transfer (Klein et al., 2000).

Bacitracin has been used both as growth promoter and in human and veterinary medicine. Low levels of resistance have been described in animal-derived isolates, especially of *Enterococcus* species (Butaye et al., 2003).

The remaining antibiotics listed in Table 1 (aminoglycosides, beta-lactams, lincosamides, quinolones, rifamycins, sulfonamides, chloramphenicol, and fusidic acid) have been employed in studies aimed at selecting foodborne AR strains of lactobacilli and lactococci, but their use as growth promoters was never reported. The corresponding AR most likely arose in environmental bacteria through selection due to improper use in human and veterinary medicine, although it cannot be excluded that such AR bacteria originated/evolved from soil-dwelling antibiotic producers, harboring AR genes for self-protection (D'Costa et al., 2006). Among them, aminoglycosides and beta-lactams are of particular interest for this review, as their corresponding AR determinants have been described in both lactobacilli and lactococci (Tables 2, 3) (Mathur and Singh, 2005; Ammor et al., 2007).

**Table 3:** **Foodborne *Lactococcus* species reported to carry AR genes and sources of isolation**.

**Species**	**Food sources**	**Resistance gene(s)**	**Association with mobile elements**	**Horizontal transfer**	**References**
*Lc. lactis*	Dairy	*tet*(M)	Tn*916*	yes	Florez et al., 2008
		*tet*(M)	nd^a^	nd	Toomey et al., 2009
		*erm*(B)	nd	nd	
		*tet*(M)	Tn*916*	yes	Boguslawska et al., 2009
		*tet*(M)	Tn*916*	yes	Devirgiliis et al., 2010
		*tet*(S), *erm*(B)	nd	nd	
		*tet*(M)	nd	nd	Toomey et al., 2010
		*dfr*A	nd	nd	Liu et al., 2009
*Lc. garviae*	Dairy	*tet*(M)	nd	nd	Walther et al., 2008
		*tet*(S)	nd	nd	
		*erm*(B)	nd	nd	
		*mdt*(A)	nd	nd	Walther et al., 2008
		*tet*(M)	Tn*916*	nd	Fortina et al., 2007
		*tet*(S)	nd	nd	
		*tet*(M)	nd	nd	Fernandez et al., 2010
		*tet*(M)	Tn6086	yes	Florez et al., 2012

a not determined.

## Antibiotic resistance in foodborne *Lactobacillus* species

A comprehensive analysis of recent publications dealing with foodborne AR lactobacilli (ARLb) was performed by browsing the PubMed database for articles published in the past 5 years. The query “Lactobacillus antibiotic resistance” retrieved about 200 articles, 30 of which were related to fermented food isolates. Half of them described at least one AR gene in the abstract and were included in the meta-analysis. The overall emerging picture, summarized in Table 2, shows that a growing number of foodborne *Lactobacillus* species has been reported to carry one or more AR genes, although the association of such genes with mobile elements as well as their possible horizontal transfer were not always investigated. The most common antibiotics employed for selection of ARLb in these studies were tetracycline and erythromycin, followed by chloramphenicol, streptomycin, ampicillin, vancomycin, and clindamycin (Table 1). Each of these antimicrobials belongs to a specific pharmacological class, and the overall information from these studies covers most of the known mechanisms of action of antibiotics (i.e., protein synthesis and cell wall assembly). Most studies were conducted employing culture-dependent phenotypic assays, followed by PCR-based detection of AR genes, while in few cases additional methodologies were used including Southern blotting, microarray assays and real time PCR (Ammor et al., 2008a,b; Devirgiliis et al., 2009; Egervarn et al., 2009). Altogether, the results from these recent studies confirm the prevalence of tetracycline and erythromycin resistance genes in lactobacilli, with *tet*(M) and *erm*(B) representing the most widespread resistance determinants (Table 2 and references therein). Moreover, these two genes were often reported to occur in genetic linkage, as in *L. paracasei* (Huys et al., 2008; Comunian et al., 2010), *L. plantarum* and *L. salivarius* (Nawaz et al., 2011). Simultaneous presence of *tet*(W) and *erm*(B) was also described in *L. paracasei* (Huys et al., 2008; Comunian et al., 2010), but their possible genetic association was not further investigated. Other genes found to confer resistance to tetracycline and erythromycin in lactobacilli were *tet*(S), (W), (K), (L), (O), *erm*(C), and *msr*(C). The latter was first described in *L. fermentum* (Thumu and Halami, 2012).

Two articles report the presence of aminoglycoside resistance genes in *L. casei* (Ouoba et al., 2008) and in *L. delbrueckii* subs *bulgaricus* (Zhou et al., 2012). This latter case represents the first example of occurrence of the *aph*(3′)-IIIa and *ant*(6) genes in *L. delbrueckii*, conferring resistance to kanamycin and streptomycin, respectively. Association with mobile elements, such as plasmids, transposons or IS is crucial to evaluate the capability for horizontal transfer of AR genes to pathogens (van Reenen and Dicks, 2011). In the case of foodborne ARLb, however, molecular characterization of the genomic context of AR genes is often lacking. In a few articles, conjugation experiments using pathogenic recipients, such as *E. faecalis* JH2-2, is the preferred method to investigate transferability of AR genes. The most common test is represented by filter mating (Devirgiliis et al., 2009; Feld et al., 2009; Nawaz et al., 2011), but *in vivo* conjugation assays in gnotobiotic rodent models have also been reported (Feld et al., 2008). Both methodologies share some limitations in terms of risk assessment, as they cannot mimic the *in vivo* situation (i.e., the crowded microbial environment characterizing the gut and food matrices) and they may therefore under-evaluate actual transfer frequencies. Interpretation of mating experiments would therefore greatly profit from the support of molecular analysis, especially when retrieving negative transfer results.

Detailed investigation of mobile element-associated AR genes, on the other hand, has been reported in only 3 papers among those analyzed in this review: in Ammor et al. two tetracycline resistance genes, *tet*(M) and *tet*(L), co-existing in a foodborne strain of *L. sakei*, were shown to reside within a transposon-like element and a plasmid, respectively (Ammor et al., 2008b); a *tet*(M) gene carried by a tetracycline-resistant strain of *L. paracasei* of dairy origin was associated to the broad host range Tn*916* transposon, which could be transferred to *E. faecalis* in filter mating assays, although with low frequency (Devirgiliis et al., 2009); nucleotide sequence of the erythromycin resistance plasmid pLFE1 from *L. plantarum* strain M345, isolated from raw-milk cheese, revealed the presence of genes involved in conjugal transfer. Filter-mating experiments confirmed the ability of pLFE1 to be transferred to *L. rhamnosus*, *Lc. lactis*, *Listeria innocua*, *E. faecalis*, and *Listeria monocytogenes*, suggesting a broad host range (Feld et al., 2009).

One of the main gaps emerging from this meta-analysis deals with the actual titer of ARLb in specific foods, and with the corresponding risk assessment for human health. This gap can be in part attributed to the heterogeneity of the study designs. Indeed, the main objective of most studies was to detect the presence of AR genes, and when possible to characterize them at the molecular level, which did not include calculating the frequency of occurrence of ARLb within the food sample (Ammor et al., 2008a,b; Ouoba et al., 2008; Egervarn et al., 2009; Liu et al., 2009; Zonenschain et al., 2009; Nawaz et al., 2011; Thumu and Halami, 2012; Zhou et al., 2012). In other papers the principal aim of the experimental work was to evaluate the probiotic features of foodborne isolates, thus leading to the analysis of AR genes or antimicrobial susceptibility only in terms of safety aspects, i.e., with the attribution of a QPS status (Fukao et al., 2009; Zago et al., 2011). Among the few articles which analyze the occurrence of ARLb from a “food safety viewpoint,” Zonenschain et al. (2009) investigated the presence of erythromycin and tetracycline resistance genes in different *Lactobacillus* species isolated from fermented dry sausages, relating the titer of ARLb to the risk of AR gene transmission. Comparative analysis of the microbiological counts of AR isolates showed that 16/20 salami could be regarded as safe, while 4 of them could be considered borderline. However, no molecular data on the association of AR genes with mobile elements was provided (Zonenschain et al., 2009).

Comunian et al. (2010) considered the cause-effect relationship between spread of antibiotic resistance in foodborne bacteria and antibiotic use in animal farming. The Authors reported a comparative analysis of 121 strains of *L. paracasei* isolated from Italian dairy and meat products manufactured in different geographical regions, in terms of resistance/susceptibility to tetracycline and erythromycin. The majority of susceptible *L. paracasei* strains originated from cheeses produced in a region where livestock are traditionally pastured, and no systematic use of antibiotics as growth promoters had been carried out over the years, while the highest number of resistant strains, shown to harbor *tet*(M), *tet*(W), and/or *erm*(B), was detected in fermented meat and cheeses from areas where more intensive practices had been applied in animal husbandry (Comunian et al., 2010); our laboratory previously reported phenotypic characterization of tetracycline, erythromycin, and kanamycin resistance in 500 LAB isolated from raw materials and final products sampled along the manufacturing process of a traditional Italian cheese, Mozzarella di Bufala Campana (MBC). AR genes were identified almost exclusively in bacteria isolated from the raw, unprocessed substrates, while the final, marketed products did not contain phenotypically resistant LAB, suggesting that the procedures adopted in the making of MBC operate a negative selection against those components of the fermenting microflora that most frequently harbor AR genes (Devirgiliis et al., 2008).

To summarize these results, we can calculate the frequency of phenotypically ARLb with respect to the total number of isolates reported in the studies allowing such extrapolation: among 22 dairy *L. plantarum*, no resistant isolates could be recovered to the antibiotics tetracycline, erythromycin, streptomycin, vancomycin, clindamycin, and chloramphenicol (Ammor et al., 2008a); analysis of 18 *L. delbrueckii* bulgaricus revealed 7 AR to the antibiotics tetracycline, kanamycin, and streptomycin (Zhou et al., 2012); Nawaz et al. reported that out of 73 foodborne Lb, tested against a panel of 14 antibiotics, 19 resulted AR (Nawaz et al., 2011), while no resistant isolates could be found among 11 Lb tested against a panel of 24 antibiotics (Ouoba et al., 2008); evaluation of resistance to the antibiotics erythromycin, tetracycline, streptomycin, ampicillin, clindamycin, and gentamycin in 115 *L. paracasei* isolates, corresponding to 66 rep-groups, resulted in the detection of 3 tetracycline and erythromycin resistant strains (Huys et al., 2008); Zago et al. found 2 tetracycline-resistant *L. plantarum* within 27 strains tested for resistance to tetracycline, erythromycin, gentamycin, and chloramphenicol (Zago et al., 2011). Overall, these numbers add to 31 ARLb in a total of 217 isolates, suggesting that the frequency of antibiotic resistance in lactobacilli is quite low in the majority of foods. A major limitation in comparing these studies stems, however, from the different methodologies employed, as well as from the different panels of antibiotics tested. Moreover, no information is provided in several cases on the presence/absence of the genes conferring phenotypic AR. Whether we can use these frequencies to define a risk range in the absence of molecular and functional data still remains therefore an open question.

## Antibiotic resistance in foodborne *Lactococcus* species

The genus *Lactococcus* includes seven different species (Odamaki et al., 2011), but only *Lactococcus lactis* subs. *lactis* and *Lc. lactis* subs. *cremoris* are involved in technological food processing. Like other LAB, lactococci can acquire antibiotic resistance under selective pressure, can survive antimicrobial treatments and consequently act as reservoir for AR genes transmissible to other bacteria. Several studies reported the susceptibility of *Lc. lactis* to Gram-positive spectrum antibiotics (erythromycin, lincomycin, vancomycin, novobiomicin, teicoplanin), to beta-lactams and to some broad-spectrum antibiotics (rifampicin, chloramphenicol, spectinomycin). On the other hand, most lactococcal species display intrinsic resistance to metronidazole, trimethoprim, and cefoxitin, and to the aminoglycosides gentamicin and kanamycin (Katla et al., 2001; Florez et al., 2005).

Although *Lc. lactis* has not yet received the acknowledgement of probiotic species, due to its low capability to colonize the human GI tract (Watterlot et al., 2010), increasing evidences point to its possible role in probiotic supplements (Casalta and Montel, 2008). This aspect, together with the successful use of several strains as dairy starters, could explain the emerging interest in considering the problem of AR also in this genus. The query “*Lactococcus* antibiotic resistance” performed for the present review in the PubMed database, and narrowed to the last 5 years, yielded about 70 articles, 10 of which describe AR species employed in food processing. Table 3 summarizes major findings from these studies, which are briefly described in the following text. As for the *Lactobacillus* genus, most of the analyzed papers applied culture-dependent phenotypic methods, and the corresponding AR genes were detected by PCR. Only few articles took advantage of supplementary assays, such as Southern blotting (Florez et al., 2008; Devirgiliis et al., 2010), microarray, and RT-PCR (Walther et al., 2008).

Florez et al. reported molecular characterization of tetracycline resistance in two *Lc. lactis* strains isolated from an artisanal starter-free cheese, which revealed the presence of the *tet*(M) gene carried by a functional Tn*916* transposon, inserted into a resident plasmid of the parental tetracycline-susceptible strain (Florez et al., 2008). The presence of *tet*(M) has not been as frequently documented in lactococci as in other LAB, such as *E. faecalis* (Hummel et al., 2007; Rizzotti et al., 2009) and *Lactobacillus* species (Gevers et al., 2003; Devirgiliis et al., 2009).

A *Lactococcus* sp. strain resistant to cloxacillin and cephalexin was found in a study aimed at assessing antibiotic tolerance of LAB in traditionally fermented Indian foods, although the presence of the corresponding AR genes was not investigated. The strain was sensitive to 13 other antibiotics, including the most representative ones among aminoglycosides, beta-lactams, cephalosporins, chloramphenicol, glycopeptides, lincosamides, macrolides, and tetracyclines (Thokchom and Joshi, 2012). In another study, Ge *et al* highlighted low level of AR among naturally occurring and starter LAB isolates from fermented dairy products from Maryland (U.S). In their study, the effect of 8 antimicrobial agents (ampicillin, ciprofloxacin, clindamycin, erythromycin, gentamicin, imipenem, tetracycline, and vancomycin) was determined, but no *Lactococcus* isolates showed phenotypic AR (Ge et al., 2007). On the contrary, rifampicin resistant lactococcal isolates from commercial products was described by Liu et al. (2009). One of the strains also carried *dfr*A, encoding a drug resistant dihydrofolate reductase (DHFR) enzyme associated with trimethoprim resistance. Interestingly, the *Lactococcus dfrA* gene is carried by the Tn*4003* transposon described in *Staphylococcus aureus*, thus indicating the probable route of transmission. However, no information on the possible genetic linkage between the two AR phenotypes was provided (Liu et al., 2009).

A phenotypic resistance screen toward 6 common antibiotics (ampicillin, chloramphenicol, erytromycin, streptomycin, tetracycline, and vancomycin) in *Lc. lactis* was reported by Toomey *et al*. In this study, 2 *Lc. lactis subs. lactis* strains and one *Lc. lactis subs. cremoris*, isolated from Irish pork and beef abattoirs, showed resistance to streptomycin. In the same study, the genetic basis of the phenotypic resistance was investigated by PCR, but no amplicons corresponding to any of the streptomycin resistance genes *strA*, *strB*, *aadA*, and *aadE* were detected (Toomey et al., 2010). As previously discussed for lactobacilli, some of the selected articles report investigation of HGT through conjugation assays. A study by Toomey et al. demonstrated that different LAB strains containing the *tet*(M) and *erm*(B) resistance genes, could transfer them to other bacteria using *in vitro* (filter mating) and *in vivo* techniques (rumen and alfa alfa sprout models). In the *in vitro* test, the highest transfer frequency among four LAB mating pairs was observed between 2 strains of *Lc. lactis* (donor SH4174, recipient BU-2-60). On the contrary, lower transfer frequencies were observed using the same LAB mating pairs in the *in vivo* test, both in rumen and in the alfalfa model, (Toomey et al., 2009). Low transfer frequencies were also presented by Bogulslawka et al., who demonstrated the ability of *Lc. lactis* isolates from Polish raw milk, to transfer the *tet*(M) determinant to *Lc. lactis* BU-2-60 and *E. faecalis* JH2-2 both *in vitro* and *in vivo*, although in this case with similar frequencies. Strains showing the highest transfer frequency were used to confirm their ability to transfer *tet*(M) to *E. faecalis* JH2-2 in the GI tract of germ-free rats (Boguslawska et al., 2009). Our laboratory has described the presence of *Lc. lactis* strains resistant to erythromycin and/or tetracycline, isolated from raw milk and natural whey samples used for MBC production. The tetracycline resistant isolates were shown to harbor a *tet*(M) gene carried by a plasmid, while the double resistant strains were shown to contain plasmid borne, genetically linked *tet*(S) and *erm*(B) genes. Filter mating experiments demonstrated horizontal transfer to *E. faecalis* JH2-2 only in the case of the *tet*(M) gene (Devirgiliis et al., 2010). Molecular analysis of the *tet*(S), *erm*(B)-containing plasmid confirmed the absence of conjugative elements promoting HGT (Devirgiliis et al., manuscript in preparation). Finally, the ability of *Lactococcus* to act as recipient in conjugal transfer experiments using a *Lactobacillus* donor, was demonstrated by Toomey et al., using a *tet*(M) determinant characterized in *L*. *plantarum*, which was successfully transferred to *Lc. lactis* strain BU-2-60 (Toomey et al., 2010).

While *Lc. lactis* subs *lactis* and *Lc. lactis* subp *cremoris* are non pathogenic and used in starter cultures for dairy products, *Lactococcus garviae* is a serious fish pathogen, and also causes mastitis in cows (Eyngor et al., 2004; Pitkala et al., 2004). However, *Lc. garviae* strains from dairy sources have been shown to be free of virulence determinants (i.e., hemolysin and gelatinase), suggesting that *Lc. garviae* of dairy origin are unconnected to the pathogenic strains (Foschino et al., 2008). This species was isolated from raw milk as well as from artisanal cheese (Casalta and Montel, 2008). The activity of *Lc*. *garviae* strains in dairy fermentations seems to contribute to the final sensory features (Fernandez et al., 2010), and no evidence was ever reported of an association between raw milk cheese consumption and human disease. Walther et al. reported the presence of AR genes in *Lc. lactis* and *Lc. garviae* strains isolated from raw milk, tested for susceptibility to 17 antibiotics. Most of the *Lc. garviae* strains showed phenotypic resistance to tetracycline and harbored *tet*(S) and *tet*(M) determinants. The Authors also report phenotypic resistance to clindamycin, erythromycin, streptomycin, and nitrofurantoin. In particular, all erythromycin resistant isolates were shown to harbor the *erm*(B) gene. The multidrug transporter *mdt*(A) was also detected in this work for the first time in *Lc. garviae*. *mdt*(A) confers resistance to macrolides, lincosamides, streptogramins, and tetracycline and it had previously been described only in *Lc. lactis* (Walther et al., 2008). A previous safety investigation by Fortina et al. revealed strains of *Lc. garviae* of dairy origin moderately resistant to kanamycin, as well as some tetracycline resistant biotypes harboring *tet*(M) and *tet*(S) (Fortina et al., 2007). Further testing in dairy strains of *Lc. garviae* against 14 antibiotics was carried out by Fernandez et al. (2010). Overall, 5 isolates showed phenotypic resistance associated to the presence of *tet*(M). More recently, Florez et al. released the draft genome sequence of *Lc. garviae* strain IPLA31405, isolated from raw milk employed in artisanal Spanish cheese production. Genome analysis revealed the presence of a *tet*(M) gene harbored by a transposon highly similar to conjugative *Tn*6086 from *E. faecalis* (Florez et al., 2012).

## Conclusions and future trends

**Figure 1:**
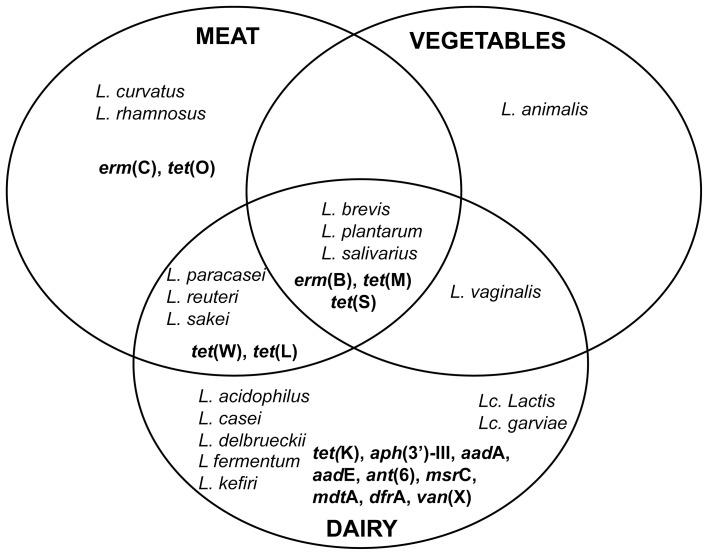
**Eulero-Venn diagram representing the distribution of ARLb and ARLc, as well as of the AR genes, in the different food sources (dairy, meat, vegetable).** AR genes are indicated in bold.

The AR Lb and Lc species detected in the 3 main sectors of fermented foods, as well as the corresponding AR genes identified in the above described studies, are graphically summarized in Figure 1. The number of species and AR genes occurring in dairy foods clearly outnumbers those detected in meat and vegetable sources. This is not surprising, when considering that the dairy sector comprises the vast majority of fermented foods and it is therefore likely to have been most intensively investigated. As for the AR genes identified, those conferring resistance to tetracycline or erythromycin are present in all food sources. *tet*(M) and *erm*(B), in particular, are confirmed as the most frequently identified and best characterized resistance genes also in terms of their genomic context and horizontal transferability. Our survey of the recent literature also appears to reflect the more general overview emerging from articles published in the past two decades, and previously reviewed by others (Mathur and Singh, 2005; Ammor et al., 2007).

The overall picture emerging from the available studies hereto described in foodborne lactobacilli and lactococci points at low frequency of occurrence of AR determinants, with even lower potential for horizontal transmission to pathogens or opportunistic pathogens, when tested in conjugation experiments. No dramatic changes seem to have occurred in foodborne AR after the EU-wide ban of antibiotics as growth promoters in livestock, and it would be tempting to conclude that consumption of fermented foods poses no real health risks in terms of AR spread to human pathogens. However, given the reported increase in pathogenic AR clinical isolates (Bush et al., 2011; Landers et al., 2012), the wide spectrum of environmental reservoirs of AR commensals (food, water, soil) (Marshall et al., 2009), and taking into account the high frequency of HGT that is known to occur within bacterial communities (van Reenen and Dicks, 2011), the real frequency of foodborne AR bacteria might be underestimated. As previously discussed, this can be partly attributed to the heterogeneity in study designs, with a great variation in the numbers and panels of antibiotics tested, while molecular analysis does not always accompany the evidence of phenotypic resistance in foodborne isolates. The mostly non-pathogenic nature of Gram-positive LAB has led to under-evaluation of the importance of characterizing the genetics of newly identified AR genes but, when carried out, it often reveals association with known conjugative transposons or IS, as well as evidence of HGT of intervening genomic sequences (Roberts and Mullany, 2011). Identification of mobile elements in the genomic context of AR genes is thus a crucial factor for evaluating the corresponding risk of HGT. The available assays to test transferability of the identified genes also pose some limitations at present, as they do not mimick the *in vivo* conditions. As shown by molecular characterization of vancomycin resistant clinical isolates of *Staphylococcus aureus*, transfer of AR genes from a commensal reservoir to opportunistic pathogens such as enterococci is the first step toward AR transmission to pathogens (Levy and Marshall, 2004). On these premises, it is understandable that the EFSA panel of scientific experts recently recommended that AR LAB species should be proven absent from animal feed and human supplements (EFSA-FEEDAP, 2012). Their expert opinion stems from a “precautionary principle” that is always necessary when insufficient scientific information is available for risk assessment.

Several questions, in our view, remain open, which impair reliable evaluation of the potential risk for human health, especially in relation to the real titer of ARLb and ARLc in specific foods, as well as on the potential transferability of the corresponding genetic determinants to human pathogens. A quantitative answer to this question would be of special relevance to contribute to a definition of the upper limits of “AR bacterial contamination” of foods that can prevent horizontal transfer of AR genes to resident gut microbiota components. Analysis of the human gut microbiota resistome is still in progress, and a comprehensive overview of AR gene distribution in this ecosystem is not yet available (Penders et al., 2013 and references therein). However, a recent metagenome-wide analysis, performed on a large cohort of human gut microbiota revealed predominance of tetracycline resistance genes (Hu et al., 2013), which appears to correlate with the prevalence of such AR determinants in foodborne LAB.

We cannot ignore that, although scientifically sound and extremely important, the information on AR in foodborne Lb and Lc emerging from the growing body of literature is still fragmentary, and profoundly affected by study designs, pointing at some knowledge gaps that need to be filled. A general issue that should be confronted is the need for methodological standardization, which is also necessary to overcome the scattered information on the genomic context of AR genes as well as on their transfer efficiency that is in turn strictly dependent on the flanking genomic structure. This probably requires meeting the experimental challenge of setting up new reliable methodologies, mimicking the actual *in vivo* conditions more closely. Genetic exchanges in bacteria are more prone to occur in crowded environments, such as the GI tract and fermented foods. One possibility would be therefore to develop food matrix and animal gut models to test inter- and intra-species conjugation within a densely populated bacterial environment, to be used with DNA-based, culture-independent, metagenomic approaches as already applied to study complex microbiota in soil or oral environments. Furthermore, a systematic and datasharing approach appears necessary at this stage to implement both the surveillance and mechanistic (HGT) studies (Bush et al., 2011; Gonzalez-Zorn and Escudero, 2012). Efforts in this direction have already been undertaken: the US-based ROAR Network (Reservoirs Of Antibiotic Resistance) as well as similar ongoing and past initiatives have created databases collecting studies and corresponding information on resistance genes and their host bacterial species (Levy and Marshall, 2004). The European Commission presently funds several projects on antimicrobial resistance and spread, mainly through its Health Programme, and has established a transatlantic task force on AR that recently published recommendations for future collaboration between the US and EU on this topic (TATFAR Report, 2011—http://ecdc.europa.eu/en/activities/diseaseprogrammes/tatfar/documents/210911_tatfar_report.pdf).

The 2006 EU-wide ban of the use of antibiotics as growth promoters has triggered an intense debate concerning the usefulness of this type of measures to effectively counteract the environmental increase and dissemination of AR bacteria (Phillips, 2007; Marshall et al., 2009; Gonzalez-Zorn and Escudero, 2012). Undoubtely, this European action has not been followed by similar bans in other countries. However, worldwide consensus has been reached on the fact that AR spread in the microbial world and the associated dramatic increase in AR bacterial infections currently represent a serious threat to human health (Levy and Marshall, 2004; Gonzalez-Zorn and Escudero, 2012). Given the multifactorial nature of the problem, the different policies on antibiotic use in different parts of the world, and the present limitations in scientific knowledge on this issue, the most effective strategy to control AR spread in bacteria should rely on multifaceted approaches, as proposed by Bush et al. (2011). Surveillance and mechanistic studies on foodborne AR LAB, among which lactobacilli and lactococci, could also greatly profit from coordination, standardization and datasharing to construct a more comprehensive and reliable picture of the actual risk of transmission of AR genes to pathogens through the food chain.

### Conflict of interest statement

The authors declare that the research was conducted in the absence of any commercial or financial relationships that could be construed as a potential conflict of interest.
